# Caring or daring? Exploring the impact of facial masculinity/femininity and gender category information on first impressions

**DOI:** 10.1371/journal.pone.0181306

**Published:** 2017-10-12

**Authors:** Mirella Walker, Michaela Wänke

**Affiliations:** 1 University of Basel, Basel, Switzerland; 2 University of Mannheim, Mannheim, Germany; University of Chicago, UNITED STATES

## Abstract

In two studies we disentangled and systematically investigated the impact of subtle facial cues to masculinity/femininity and gender category information on first impressions. Participants judged the same unambiguously male and female target persons–either with masculine or feminine facial features slightly enhanced–regarding stereotypically masculine (i.e., competence) and feminine (i.e., warmth) personality traits. Results of both studies showed a strong effect of facial masculinity/femininity: Masculine-looking persons were seen as colder and more competent than feminine-looking persons. This effect of facial masculinity/femininity was not only found for typical (i.e., masculine-looking men and feminine-looking women) and atypical (i.e., masculine-looking women and feminine-looking men) category members; it was even found to be more pronounced for atypical than for typical category members. This finding reveals that comparing atypical members to the group prototype results in pronounced effects of facial masculinity/femininity. These contrast effects for atypical members predominate assimilation effects for typical members. Intriguingly, very subtle facial cues to masculinity/femininity strongly guide first impressions and may have more impact than the gender category.

## Introduction

When we see a person for the very first time, we instantly and spontaneously draw inferences from that person’s face. These inferences pertain to the person’s group memberships or social category (e.g., [1]) as well as to their personality (e.g., [2]). Both types of inferences share (at least) two important characteristics indicating that they are not independent from each other. Firstly, facial masculinity or femininity plays a crucial role both in categorizing a person as male or female [3–7] and in the ascription of personality [8]. A feminine-looking woman, for example, is not only more easily categorized as a woman [3,4], she is also more likely perceived as warm–a stereotypical female trait–than a masculine-looking woman [8]. Secondly, both inferences are related to processes of stereotyping. On the one hand, classifying a person as female activates consensual beliefs about the characteristics of women (e.g., women are warm [9]), which might then lead to the ascription of the personality traits perceived as stereotypic for the group to the group members (i.e., category-based gender stereotyping). Spontaneous, automatic personality inferences, on the other hand, are the result of overgeneralization effects, among which sex overgeneralization is a prominent one [10]. If the facial features of an individual resemble the facial features that are perceived as typical for a specific stereotyped group (e.g., women), the individual is likely perceived to possess the personality traits associated with the stereotyped group (e.g., warmth), irrespective of the gender of the individual (i.e., direct feature-trait associations [11]).

The aim of this paper is to systematically disentangle gender category cueing information from facial masculinity/femininity to systematically investigate the impact of category-based gender stereotyping and feature-trait associations in direct comparison. It is important to note that although facial masculinity/femininity overlaps with gender category, insofar as on average men have more masculine-looking and women have more feminine-looking faces, there is also variance within each category. Some women are more masculine-looking than others, while some men are more feminine-looking than others. It is on the atypical exemplars where different models make different predictions: Do category-based gender stereotyping and feature-trait associations work simultaneously causing stronger differences between feminine-looking women and masculine-looking men (typical category exemplars) than between masculine-looking women and feminine-looking men (atypical category exemplars)? Or, in line with the inclusion/exclusion framework [12], are masculine-looking women and feminine-looking men contrasted against the stereotype of their gender category resulting in stronger effects for atypical than for typical exemplars?

### Disentangling facial masculinity/femininity from gender category information

To systematically investigate the interplay of category-based gender stereotyping and feature-trait associations we first had to disentangle facial masculinity/femininity from gender category information. Every face has more or less unique facial characteristics. Some of these characteristics can be described in terms of single features (e.g., full lips, a roundish chin), whereas others can be described in terms of the configuration of features (e.g., close-set eyes). There is a lot of evidence in the face perception literature that featural information is more relevant for the categorization of individuals as either male or female, whereas configural information is more relevant to identify a person [4,13]. However, there is also evidence that facial information alone is often not sufficient for gender categorization [14,15]. Especially women lacking extra-facial features, such as hairstyle, jewelry, or clothing tend to be misclassified as men. Often extra-facial styling information is needed to classify a person as either male or female. Among these, the hairstyle has been shown to be the best category-cueing feature [4,13].

Whereas extra-facial hairstyle information is a potent cue for gender categorization, inner facial masculinity/femininity has been shown to impact spontaneous impression formation processes (e.g., [16]). Different approaches have been developed to systematically model masculinity/femininity in faces in the last 20 years [15,17,18]. Using one of these approaches, namely the morphable model approach [17], we calculated a masculinity/femininity vector by determining the direction between the average male and the average female face within a statistical face space based on 100 male and 100 female faces [19]. This method is fully data-driven or bottom-up in the sense that the masculinity/femininity vector depicts the information that discriminates between the average male and female face [20]. Because both features (size of eyes, fullness of mouth) as well as configuration (proximity among the features) differ between male and female faces, our conceptualization of inner-facial masculinity/femininity consists of both featural and configural information. This information–inner-facial masculinity/femininity–was then systematically modeled in the stimulus persons’ faces to make them appear more or less masculine or feminine [16,17,21]. In this paper we–for the first time–modeled facial masculinity/femininity using up-to date computer graphics methods to manipulate real face photographs in a controlled way and with natural-looking results [20]. We created feminine- and masculine-looking versions of the same unambiguously male and female target persons to systematically investigate the interplay of extra-facial category cueing information and masculinity/femininity in direct comparison.

### How gender category and gendered facial information impact personality inferences

From a mere category-based gender stereotyping perspective, one would expect that based on extra-facial category-cueing information (i.e., hairstyle) individuals were classified as males or females. By classifying a person as male or female, an association between the person and a gender category is established. This process likely activates the content of the respective gender stereotype that subsequently can be used to draw inferences about the person [22]. As a result, the judgment of the person will be assimilated towards the stereotype. Consequently, category-based gender stereotyping would predict that persons classified as men are ascribed stereotypically masculine personality traits, whereas persons classified as women are ascribed stereotypically feminine personality traits. In line with the Stereotype Content Model we will refer to these stereotypical traits as warmth (stereotypically feminine traits) and competence (stereotypically masculine traits), respectively [23].

From the perspective of feature-trait associations, one would expect that the facial masculinity/femininity–independent of category membership–was directly linked to the perception of stereotypically masculine or feminine personality traits. (Note that the term feature-trait associations refers to the bottom-up process of directly linking facial information with personality traits [11]; it does not imply that configural facial information is not relevant for this process.) These expectations find support by research from two domains, namely the domain of cultural stereotyping and the domain of impression formation from faces. On the one hand, evidence from research on cultural stereotyping has shown that the extent of Afrocentric features in a target was positively correlated with judgments stereotypical to African-Americans [11,24,25]. This was true for African-American and White targets indicating that facial features affected the judgment independent of ethnic category. Most importantly, the effect was not mediated by category accessibility [24]. On the other hand, evidence from face-based person perception research revealed that when seeing a stranger, individuals automatically locate him or her in a 2D coordinate system spanned by the dimensions trustworthiness and dominance [8]. Work on spontaneous personality inferences from faces has shown associations between facial femininity and trustworthiness as well as facial masculinity and dominance [8]. Consequently, there is a lot of evidence for the feature-trait association account predicting that masculine-looking persons are spontaneously ascribed more competence and less warmth than feminine-looking persons.

Whereas both the category-based stereotyping and the feature-trait association account make identical predictions for feminine-looking women and masculine-looking men (typical exemplars), they make opposite predictions for masculine-looking women and feminine-looking men (atypical exemplars). For typical exemplars both models predict that masculine-looking men are ascribed more competence and less warmth than feminine-looking women either because they are included in the respective gender category and consequently assimilated towards that gender category or because of strong associations between their individual facial features and the personality dimensions trustworthiness and dominance. For atypical exemplars, the implications of category membership and individual features point in opposite directions. If the judgment is derived from the category membership, assimilation to the gender category is expected. Thus, feminine-looking men would be ascribed *more competence* and *less warmth* than masculine-looking women. If, however, facial features would be directly linked to the respective personality dimensions, feminine-looking men would be ascribed *less competence* and *more warmth* than masculine-looking women.

When comparing the two models there are at least two reasons to believe that if both category and feature information is available, feature-trait associations should override category-trait associations. First, there is evidence that stereotypes are not likely to affect personality judgments, if diagnostic individuating information about the target is available [26]. And we know from the ecological theory of face perception (e.g., [10]) that individuals regard certain facial information (e.g., the information associated with gender) as diagnostic for certain behavioral affordances and personality traits. Because the facial appearance of a target person provides a perceiver with more detailed, concrete, and individuating information about a target person than mere category membership and because perceivers rely on facial information to build first impressions, feature information is likely to outweigh category information in the process of impression formation.

Second, although both category-based stereotyping and direct feature-trait associations often work implicitly and automatically (e.g., [24] for feature-trait associations; e.g., [27] for stereotyping), individuals might be more aware that they have a tendency to simplify the world around them by classifying individuals into categories and use stereotypic information about these categories to describe the individuals than they are aware that they use facial information to infer personality information. Therefore, they are more likely to control for the effects of category information than for the effects of feature information, particularly if cues are subtle [25].

Importantly, however, a feature-based account does not necessarily imply that the respective category will be ignored and has no influence. It has been shown that even gender-atypical faces of men and women activate the matching gender category [28]. Participants in a mouse tracking study had to associate typical and atypical men and women with either the male or the female gender stereotype. Although mouse trajectories for masculine-looking women and feminine-looking men showed more attraction towards the male and female gender stereotype respectively, as compared to the trajectories for feminine-looking women and masculine-looking men, female faces were generally associated with the female stereotype, male faces with the male stereotype [28].

How does this activated category information impact further processing of the stimulus? Research suggests that the category may provide a frame of reference for the judgment [29]; for a review, see [30]. The inclusion/exclusion model [30,31] holds that the target will be judged with reference to the category’s standard by being compared to the category prototype. Atypical members of a category are likely to be excluded from the gender category. A masculine-looking woman, for example, would not only spontaneously activate the concept of competence, but also the female gender category. Because of her facial appearance, she would be excluded from that category, for example, by subtyping her into the category of career woman [32]. This in turn would result in a contrast or a shift away from the stereotypical woman, resulting in more pronounced ascriptions of competence or stereotypically masculine personality traits.

To summarize, for atypical category exemplars (i.e., masculine-looking women and feminine-looking men) two processes are likely to co-occur: (a) feature-trait associations lead to perceptions of high levels of competence and low levels of warmth in women and vice versa for men and (b) gender category is used as a reference against which the atypical exemplar is contrasted, also leading to perceptions of high levels of competence and low levels of warmth in women and vice versa for men. Because both processes are likely to work simultaneously, we expect stronger effects for atypical than for typical exemplars. The masculine facial appearances of a woman and of a man elicit the same level of competence (and warmth) inferences due to feature-based inferences, but the woman may be judged as even more competent (and colder) than the man because she is additionally contrasted against the female prototype. From these theoretical assumptions, the following hypotheses ensue:

First, due to feature-trait associations we assume that overall masculine-looking persons are perceived as more competent and less warm than feminine-looking persons (Hypothesis 1) [11,24,25]. Second, because feature-trait associations likely override category-based stereotyping we hypothesize that masculine-looking women appear more competent and less warm than feminine-looking men (Hypothesis 2) [25]. Third, because atypical exemplars are expected to be additionally contrasted against their group prototype, the effects of facial masculinity/femininity appear more pronounced for atypical than for typical group members (Hypothesis 3) [30,31,33].

### The present studies

In both of the studies presented here we tested the three above-mentioned hypotheses by manipulating category-cueing information and facial masculinity/femininity orthogonal to each other. Participants had to judge two target persons regarding stereotypically masculine (i.e., competence) and stereotypically feminine (i.e, warmth) personality traits based on their portraits in a sequence. Because different faces are associated with different personalities, which produces considerable confound when using real face photographs, we used a data-driven statistical face model [16,17,19,21] to systematically manipulate the facial masculinity/femininity within the same faces. This approach allowed us to avoid confound and to disentangle facial masculinity/femininity information from the person and thereby also from the gender category. Using such a data-driven modeling approach [16,21] had another advantage: We did not have to a priori define which characteristics–be they featural or configural–make a face appear more masculine or feminine. This information was directly extracted from the face model.

This approach to investigate the impact of facial masculinity/femininity and gender category information is novel for different reasons. Overall, little work has been done separating category and feature information. The work that has been done was either based on material that was controlled but artificial looking or natural looking but confounded. On the one hand, Little and colleagues, for example, used very controlled stimuli with systematic variations in masculinity/femininity. However, their stimuli lack extra-facial information and thus appear somewhat artificial (e.g., [18,34]). On the other hand, Blair and colleagues, for example, in the seminal work on the role of Afrocentric facial features on criminal sentencing [11,24] used real face photographs and measured the degree of Afrocentricity. Not surprisingly, there was a considerable overlap between Afrocentric facial features and ethnic category. And potentially, the facial appearance dimension of interest (i.e., Afrocentricity) was confounded with other facial appearance information of the individual stimulus persons used.

By using novel image manipulation techniques, we were able to use facial stimuli that are at the same time controlled and natural looking. To the best of our knowledge the present studies are the first that unambiguously separate gender category and gendered facial appearance information in studying their effects on explicit ascriptions of gender-stereotypical personality traits.

### Ethics statement

The following studies were conducted in full accordance with the Ethical Guidelines of the Swiss Psychological Society (SGP-SSP) and the American Psychological Association (APA). At the time of data acquisition (before 2014) it was not possible at the University of Basel to seek ethics approval for survey studies. We did not collect any sensitive data. All questionnaires in the study were anonymous questionnaires. Moreover, we obtained consent from all participants and participants could easily withdraw from the study at any time by closing the Internet browser. After the establishment of the IRB at the Department of Psychology at the University of Basel (2014) we applied for and received IRB approval for the paradigms employed in these studies (IRB approval No. 034-15-3).

## Study 1

### Method

#### Participants and design

Participants were conveniently sampled online via digital bulletin boards and social media pages of different German-speaking Universities for a survey that took approximately 15 minutes to complete (actual duration: *M* = 13.18, *SD* = 6.92). A total of 299 participants (240 women, 58 men, 1 person did not indicate gender) took part in this online-study implemented in unipark [35]. Their mean age was 26.88 years (*SD* = 7.18). The design was a mixed 2 (facial appearance: masculine-looking vs. feminine-looking; within-participants) x 2 (target persons’ gender: male vs. female; between-participants) x 2 (rated personality dimension: competence vs. warmth; within-participants) design with the dependent variable personality judgment. A power analysis with Pangea [36] ensured that using this research design a sample size of approximately 300 with an estimated moderate to large effect size (0.5 < d < 0.8) would result in a power of .80.

#### Material

The first independent variable, namely target persons’ gender was manipulated by preselecting three male and three female faces from the FERET database [37]. The primary preselection criterion was that the three male persons had a short, masculine hairstyle and that the three female persons had a long, feminine hairstyle to unambiguously trigger the activation of the male and female gender category, respectively. Secondary preselection criteria were closed mouths, open eyes, direct gaze, neutral facial expression, and a lack of facial hair or glasses. These additional criteria were accounted for by the manipulation procedure described in the next section.

**Fig 1:**
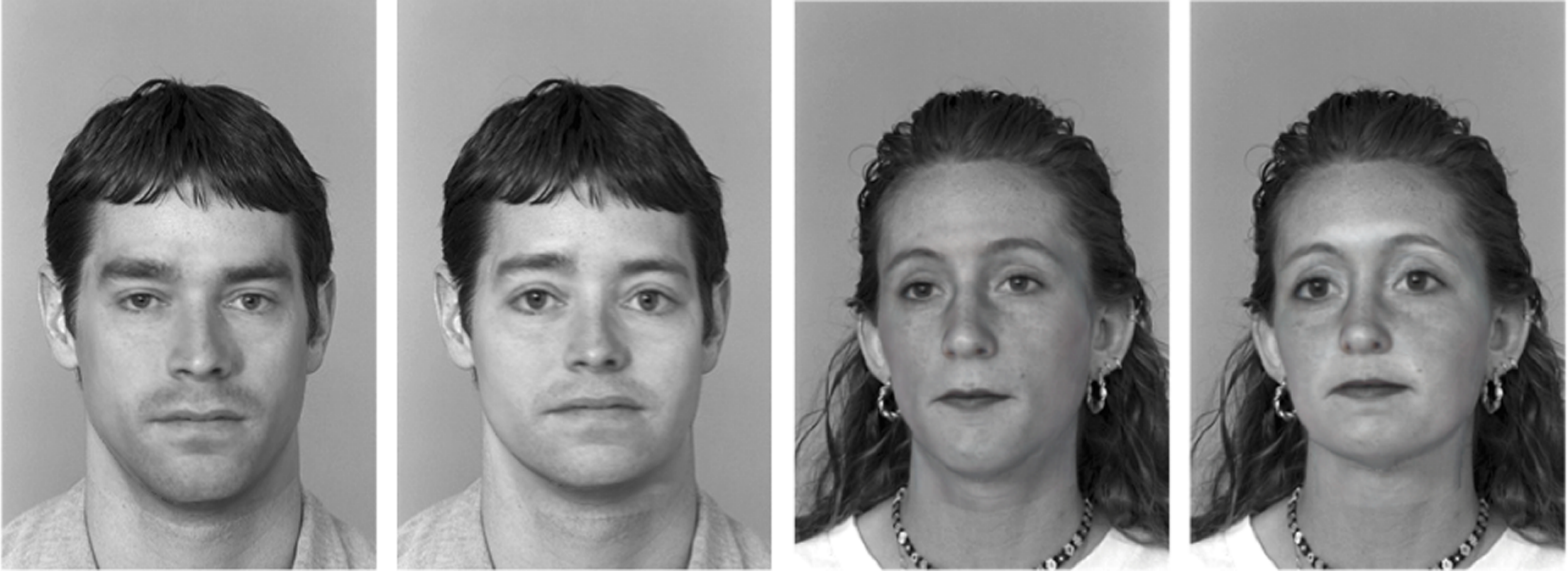
Example of stimulus material used in both studies. Note that the gender category can always clearly be inferred from the extra-facial hairstyle information, whereas the masculinity/femininity of the facial appearance is determined from inner facial featural and configural information. From left to right: Masculine-looking man, feminine-looking man, masculine-looking woman, and feminine-looking woman. The original faces (not depicted here) stem from the FERET database [37].

The second independent variable, namely facial masculinity/femininity was manipulated using a data-driven statistical modeling approach. First, the preselected faces were analyzed by actively synthesizing them with a statistical face model [19] resulting in estimations of the head structure and the texture information of the three male and three female faces. Then we generated a masculinity/femininity vector by first averaging all 100 male and all 100 female faces in our face space, respectively, and then computing the vector pointing from the average male to the average female face. This masculinity/femininity vector depicts all information, featural (e.g., bigger eyes in females, more pronounced chins in males) and configural (e.g., single features are closer to each other in female faces), that make a face appear more masculine/feminine. This masculinity/femininity vector was then used to systematically enhance and reduce facial masculinity/femininity in the six preselected faces, resulting in two new versions of all of them, namely a more masculine- and a more feminine-looking version. The resulting heads were rendered back into the original photographs, showing natural-looking variations of masculinity/femininity in all six faces (see Fig 1). Note that all faces were manipulated in the exact same way, because the same kind and the same amount of information were modeled in all of them in a very controlled way. This procedure resulted in twelve different stimuli, namely a masculine- and a feminine-looking version of all six preselected faces. For a more detailed description of the image manipulation technique, see [16,21]; for data demonstrating that these face models can successfully be transferred to novel faces even if these faces differ strongly from the faces on which the models were developed, see [38].

The twelve faces were combined in pairs of two faces each with two constraints: Facial masculinity/femininity information should be contrary and gender category information should be the same. This procedure resulted in six pairs of faces for each gender category (e.g., pair 1: male face 1 masculine/male face 2 feminine, pair 2: male face 1 masculine/male face 3 feminine, pair 3: male face 2 masculine/male face 3 feminine, pair 4: male face 1 feminine/male face 2 masculine, pair 5: male face 1 feminine/male face 3 masculine, pair 6: male face 2 feminine/male face 3 masculine).

In order to assess gender stereotypical personality inferences, we used a German version [39] of the Bem Sex Role Inventory (BSRI [40]). This questionnaire consists of 20 items measuring stereotypically masculine (e.g., leadership qualities, assertive, authoritative, resolute, confident; referred to as competent below), stereotypically feminine (e.g., softhearted, sensitive, romantic, sentimental, sincere; referred to as warm below), and gender-neutral personality traits (e.g., nervous, healthy, sad, conscientious, oblivious) each. This questionnaire has been shown to successfully measure gender stereotypes (e.g., [41,42]). The gender-neutral personality traits were used as filler items in order not to make the target of our study too obvious. The items were reframed to assess personality in the third instead of the first person perspective (i.e., “The depicted person has leadership qualities”, “The depicted person is softhearted”). These traits were rated on a 7-point Likert Scale ranging from 1 *(I strongly disagree*) to 7 (*I strongly agree*).

#### Procedure

Participants were randomly assigned to one of two conditions (i.e., target persons’ gender: male vs. female). Within each condition they were randomly assigned to one of six pairs of faces as described above. On the first web page they were welcomed and told that they were taking part in a study about impression formation. Then they were shown the first target person and judged him or her on 60 items from the BSRI. Subsequently, they were presented with the second target person and again judged him or her on 60 items from the BSRI. Order of presentation of the target persons was random. Finally, participants were asked to give some demographical information (i.e., gender, age, and mother tongue) and leave their email-addresses in order to take part in a lottery.

### Results

First, a masculinity/competence and a femininity/warmth score were built by averaging all the respective items from the BSRI. Both scores showed a high internal consistency with a Cronbach’s alpha of .94 for the masculinity scale and a Cronbach’s alpha of .89 for the femininity scale. Because each participant had to evaluate both masculinity/competence and femininity/warmth of all faces, we treat these two dimensions as two levels of a within-subjects factor personality dimension in what follows.

We analyzed our data using linear mixed models analyses including random effects for participants and faces. The advantage of this method is that it allows the generalization of results not only across participants, but also across faces. Consequently, this method allows to make inferences to future studies with different samples of participants and faces and enhances replicability of the findings [43]. To test Hypothesis 1 we used the *lme4* [44] package in *R* [45] to fit a mixed linear model with the following specifications to our data: Facial appearance (masculine-looking vs. feminine-looking; within-participants), target gender (male vs. female; between-participants), personality dimension (competence vs. warmth; within-participants), all three possible 2-way interactions, and the three-way interaction were included into the model as fixed effects, while both participants and faces were included as random effects. We aimed for a maximal linear mixed model because such models generalize best across participants and stimuli [46]. Therefore, we included a random intercept (i.e., the model allows the intercept to vary individually) and a random slope for the main effect facial appearance based on participants (i.e., the model allows the facial appearance to individually affect different participants’ judgments) and a random intercept and random slopes for facial appearance, personality dimension, and the facial appearance x personality dimension interaction based on faces (i.e., the model allows the intercept to vary individually and the facial appearance, personality dimension, and the interaction between the latter two to individually affect different faces). The reason for only including the facial appearance random slopes for participants is that we had one observation per participant for each combination of fixed factor levels [46]. Table 1 shows the explained variance by the random factors specified above.

**Table 1:** Variance explained by the random effects for participants and faces in Study 1.

Random Effects		Variance
Participants	Intercept	< .001
	Facial appearance	< .001
Faces	Intercept	.040
	Facial appearance	.104
	Personality dimension	.214
	Facial appearance * Personality dimension	.182
Residual		.709

To calculate *F*, and *p* values as in a classical linear model, we additionally used the *lmerTest* package [47] in *R* [45]. Results of the analysis with the dependent variable ascribed personality revealed one significant effect, namely the two-way interaction between facial appearance and ascribed personality, *F*(1, 3.97) = 68.42, *p* = .001. Uneven degrees of freedom result from Satterthwaite approximations. We do not present effect sizes, because no standard has been established, yet on how to include the multiple variance components from the random intercepts and slopes in the model.

Supporting our first hypothesis, masculine-looking target persons were ascribed more competence than feminine-looking target persons, *F*(1, 3.94) = 36.07, *p* = .004, whereas feminine-looking target persons were ascribed more warmth than masculine-looking target persons, *F*(1, 3.99) = 103.47, *p* < .001 (adjusted means and standard errors are presented in Table 2.). For exploratory reasons we also ran an analysis with the additional fixed factor participant gender. This analysis did not reveal any main or interaction effect including participant gender, *F*_*max*_(1, 1168.99) = 1.54, *p* = .215.

**Table 2:** Means (standard deviations) for the dependent variables ascribed competence and warmth depending on target persons’ gender and facial appearance in Study 1. The higher the mean values, the more the stimulus persons are perceived as competent and warm, respectively.

	Male target person		Female target person	
	Masculine-looking (typical men) *M (SE)*	Feminine-looking (atypical men) *M (SE)*	Feminine-looking (typical women) *M (SE)*	Masculine-looking (atypical women) *M (SE)*
Competence	4.48 (.14)	3.51 (.18)	3.60 (.18)	4.36 (.13)
Warmth	3.51 (.18)	4.18 (.15)	4.15 (.15)	3.26 (.18)

**Fig 2:**
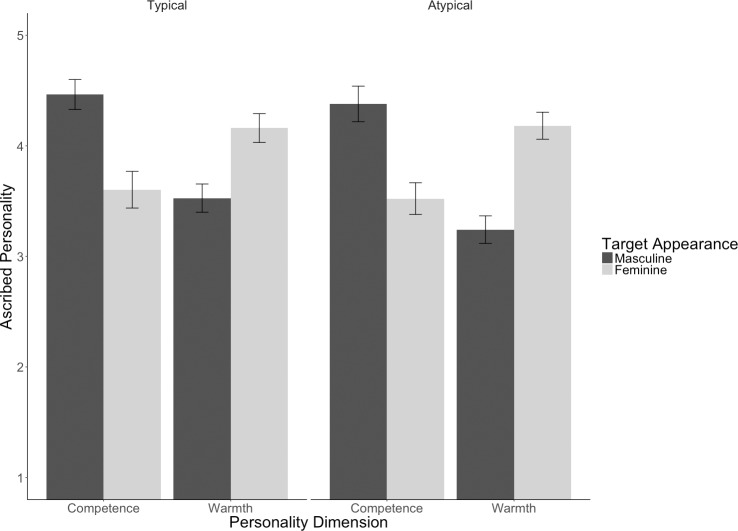
Impact of facial appearance and gender information on the ascription of competence and warmth measured with the BSRI for typical and atypical category members in Study 1. Error bars represent standard errors of the mean.

To test Hypotheses 2 and 3, we built a model with the three fixed factors ascribed personality, target gender, typicality, all three two-way interactions, and the three-way interaction effects between these as well as random intercepts for participants and random intercepts and random slopes for the main effect personality dimension based on faces. We defined the factor typicality so that masculine-looking males and feminine-looking females were coded as typical, whereas feminine-looking males and masculine-looking females were coded as atypical. Results revealed a significant main effect of typicality, *F*(1, 1182.21) = 5.07, *p* = .025. This effect, however, was qualified by a significant three way-interaction, *F*(1, 1181.71) = 281.621, *p* < .001, signaling that ascriptions of personality significantly differs between typical and atypical category exemplars. Supporting Hypothesis 2, results revealed significant effects of feature-trait associations for typical and atypical exemplars: For typical exemplars we found that masculine-looking men were ascribed more competence and less warmth than feminine-looking women, *F*(1, 4.05) = 16.32, *p* = .015. For atypical exemplars we also found that masculine looking faces (i.e., masculine looking women) were ascribed more competence and less warmth than feminine faces (i.e., feminine-looking men), *F*(1, 4.04) = 30.18, *p* = .005. However, in line with Hypothesis 3, the higher *F* value for the feature effect in the atypical than in the typical condition combined with the significant 3-way interaction reveals that the effect of facial features was much larger for atypical than for typical exemplars. Whereas feature-trait associations affect both typical and atypical category members, an additional mechanism is at work for atypical members, which likely is a contrast effect to the category prototype. The impact of target persons’ facial appearance and gender on the ascription of competence and warmth is illustrated in Fig 2.

To further investigate whether the results found followed from a direct comparison between the person in the first and second trial (i.e., a feminine-looking (wo)man presented after a masculine-looking (wo)man and vice versa) we analyzed the two trials separately. We therefore specified two models with the fixed factors facial appearance (masculine-looking vs. feminine-looking; between-participants, because we only analyzed one trial at a time), target gender (male vs. female; between-participants), personality dimension (competence vs. warmth; within-participants), all three possible two-way interactions, and the three-way interaction for both trials separately. Again, we included random intercepts for participants and stimuli as well as random slopes for facial appearance and gender for the different faces. For both trials separately, we could replicate the finding that masculine-looking persons were ascribed more competence and less warmth than feminine-looking persons, *F*_*Trial1*_(1, 584.61) = 100.51, *p* < .001 and *F*_*Trial2*_(1, 585.43) = 201.43, *p* < .001, respectively. We also specified the linear mixed models with the three fixed factors facial appearance, typicality, and ascribed personality and all four possible interaction effects as well as the random effects specified above. The three-way interaction between masculinity/femininity, typicality, and ascribed personality did not reach significance in any of the two trials, *F*_*Trial1*_(1, 4.05) = 1.87, *p* = .242 and *F*_*Trial2*_(1, 4) < 1, respectively.

### Discussion

In line with our first hypothesis, masculine-looking target persons were ascribed more competence and less warmth than feminine-looking target persons. This effect of facial masculinity/femininity was present both in the first and second trial per participant, showing that this effect does not require a direct comparison of different persons. Moreover, we found this feature-effect both for the typical and for the atypical category members, supporting our second hypothesis. Interestingly, in line with our third hypothesis, the effect was even more pronounced for atypical than for typical category members.

These findings extend results by Freeman and Ambady [28] in two ways. First, they do not only show that atypical category members activate both gender stereotypes, but demonstrate that the stereotype activated by facial appearance is reflected in explicit personality judgments. Second, they suggest that in contrast to the stereotype activated by facial features there is no evidence for category-based stereotyping. The implications of the gender category did not weaken those of facial appearance for atypical exemplars, instead, the degree to which masculine-looking women are ascribed more competence and less warmth than feminine-looking men is higher than the degree in which masculine-looking men are ascribed more competence and less warmth than feminine-looking women. These results are in line with predictions from the inclusion/exclusion model [31] suggesting that atypical category members are contrasted against their group prototype on top of the feature effect. This finding indicates that the activated gender category is used as a frame of reference creating a stereotype reversal.

It is possible that the design of the first study may have worked against assimilation effects based on gender category. The fact that all judgments involved either only women or only men may have made the gender category less salient as a base for inferences. The first target may have rendered within-category comparisons particularly likely for the second target. In order to test the generalizability of the results we changed the design in Study 2.

## Study 2

In Study 2, both gender category and facial appearance information was manipulated within participants. Participants either saw a masculine-looking man and a feminine-looking woman (typical condition) or a feminine-looking man and a masculine-looking woman (atypical condition). By manipulating gender of the stimulus person within participants, gender category information should become more salient here than in Study 1. The main reason for Study 2 was to investigate whether under these conditions category-based gender stereotyping would occur. Moreover, we used additional scales to measure competence and warmth to test whether results were robust across different measures.

### Method

#### Participants and design

Participants were conveniently sampled via digital bulletin boards and social media pages of different German-speaking Universities for a survey that took approximately 10 minutes to complete (actual duration: *M* = 8.04, *SD* = 4.58). Ninety-eight participants (69 women, 29 men) took part in this study implemented in unipark [35]. Their mean age was 25.79 years (*SD* = 9.06). The design was a mixed 2 (facial appearance: masculine-looking vs. feminine-looking; within-participants) × 2 (typicality of category members: typical vs. atypical; between-participants) design with the dependent variables ascribed competence and warmth. Different from Study 1, this design enables analysis of the data as a function of facial appearance and typicality (or gender and typicality) but does not enable analysis of the data as a function of facial appearance and gender simultaneously. Because of the rather big mean differences obtained in Study 1, we reduced the sample size in Study 2.

#### Material

Two male and two female faces from the FERET database [37] were preselected using the same criteria as in Study 1. These faces were manipulated exactly as in Study 1.

Five items from both the masculinity and the femininity scale of the German version of the BSRI [39] were used and again reframed to assess personality in the third person perspective (i.e., the 10 examples given in Study 1). Additionally, five competence- (i.e., strong-nerved, decisive, analytic, self-reliant, determined) and five warmth-items (i.e., sociable, cooperative, caring, mediative, balanced) from a German inventory directly measuring personality in the third person perspective were used (Konstanzer Managergeschlechtsrolleninventar, KMGI [48]). All traits were rated on a 7-point Likert Scale ranging from 1 (*I strongly disagree*) to 7 (*I strongly agree*). Again, we calculated a competence or masculinity and a warmth or femininity scale by averaging the ten items (i.e., five from the BSRI and five from the KMGI) of the respective dimension.

#### Procedure

The procedure was similar to Study 1. Data were collected online. Participants were either presented with a photograph of a masculine-looking man and a feminine-looking woman (typical category exemplars) or a feminine-looking man and a masculine-looking woman (atypical category exemplars). Photographs were presented in random order.

### Results

Again, we first calculated a competence and a warmth scale by averaging the ten items (i.e., five from the BSRI and five from the KMGI) of the respective dimension. Both scales showed a high internal consistency (Cronbach’s alpha_Masc_ = .91 and Cronbach’s alpha_Fem_ = .88).

As in Study 1, we fitted a mixed linear model with ascribed personality as the dependent variable using the *lme4* [44] and the *lmerTest* package [47] in *R* [45]. Facial appearance (masculine-looking vs. feminine-looking; within-participants), typicality of category members (typical vs. atypical; between-participants), ascribed personality (competence vs. warmth; within-participants), all three two-way interactions, and the three-way interaction were included into the model as fixed effects, while both participants and faces were included as random effects. Thereby this model accounts for sampling variability both regarding participants and faces [43]. Aiming for a maximal linear mixed model [46] we included random intercepts and a random slope for the main effect facial appearance based on participants and faces. We did not include the interaction term between facial appearance and personality dimension, because the respective model did not converge. To make sure that results are nevertheless reliable we also fitted a model with random intercepts and slopes for personality dimension for participants and faces. Neither the significance levels nor the pattern of results changed. Table 3 shows the explained variance by the random factors in this model.

**Table 3:** Variance explained by the random effects for participants and faces in Study 2.

Random Effects		Variance
Participants	Intercept	.102
	Facial appearance	.014
Faces	Intercept	.003
	Facial appearance	.103
Residual		1.065

Supporting our first hypothesis and thereby replicating Study 1, we found a two way interaction between facial appearance and personality dimension, showing that masculine-looking persons are ascribed more competence and less warmth than feminine-looking persons, *F*(1, 286.34) = 47.91, *p* < .001 (see Table 4 for adjusted means and standard errors.). For exploratory reasons we also ran an analysis with the additional fixed factor participant gender. Results revealed one significant interaction effect with the factor participant gender, namely a typicality-by-participant gender interaction, *F*(1, 93.39) = 5.28, *p* = .024. On both DVs female participants gave higher ratings to typical looking persons (*M* = 3.89, *SE* = .11) than to atypical looking persons (*M* = 3.60, *SE* = .12), whereas male participants gave higher ratings to atypical looking persons (*M* = 3.87, *SE* = .17) than to typical looking persons (*M* = 3.58, *SE* = .15). However, these results should be interpreted with caution, because this effect was merely exploratory and bears theoretical derivation.

**Table 4:** Means (standard deviations) for the dependent variables ascribed competence and warmth depending on target persons’ gender and facial appearance in Study 2. The higher the mean values, the more the stimulus persons are perceived as competent and warm, respectively.

	Male target person		Female target person	
	Masculine-looking (typical men) *M (SE)*	Feminine-looking (atypical men) *M (SE)*	Feminine-looking (typical women) *M (SE)*	Masculine-looking (atypical women) *M (SE)*
Competence	4.21 (.15)	3.16 (.24)	3.72 (.24)	4.16 (.17)
Warmth	3.39 (.15)	4.14 (.24)	3.89 (.24)	3.22 (.17)

**Fig 3:**
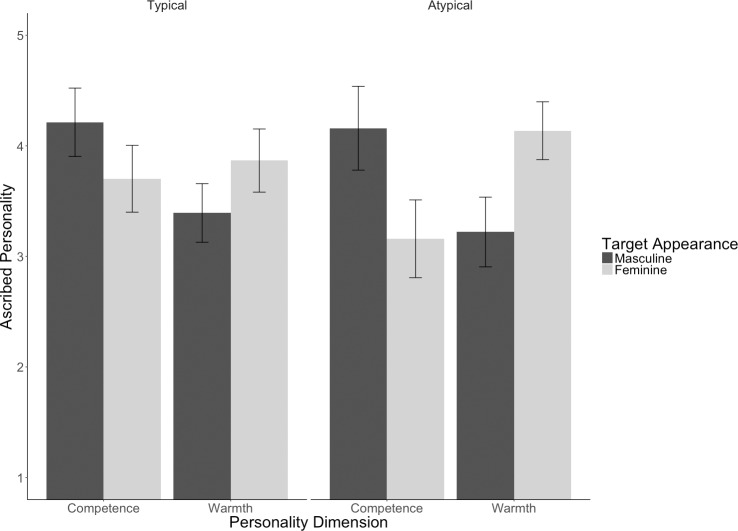
Impact of target persons’ facial appearance and gender on the ascription of competence and warmth measured with the BSRI and the KMGI for typical and atypical category members in Study 2. Bars represent errors of the means.

The two-way interaction of facial appearance and ascribed personality was qualified by the three-way interaction of facial appearance, ascribed personality, and typicality; *F*(1, 286.34) = 4.93, *p* = .027. Looking at the interaction of facial appearance and ascribed personality separately for typical and atypical exemplars reveals that in line with our second hypothesis, the effect of feature-trait associations was not only significant in the typical, *F*(1, 159) = 13.51, *p* < .001, but also in the atypical condition, *F*(1, 170) = 34.91, *p* < .001. The higher *F* value for the feature effect in the atypical than in the typical condition combined with the fact that these feature effects significantly differ from each other as indicated by the three-way interaction provide evidence for Hypothesis 3. Again, using mixed models procedures to analyze data support the notion that these results generalize across participants and stimuli. To facilitate comparisons between the results of both studies, Fig 3 visualizes the conditions as in Study 1.

Again, aiming to investigate whether the results found followed from a direct comparison between the person in the first and second trial we analyzed the two trials separately. We therefore specified two models with the fixed factors facial appearance (masculine-looking vs. feminine-looking; between-participants, because we only analyzed one trial at a time), target gender (male vs. female; between-participants), personality dimension (competence vs. warmth; within-participants), all three possible 2-way interactions, and the three-way interaction for both trials separately. Again, we included random intercepts for participants and stimuli as well as random slopes for facial appearance and gender for the different faces. For both trials separately, results revealed an interaction between facial appearance and personality dimension, thereby replicating the finding that masculine-looking persons were ascribed more competence and less warmth than feminine-looking persons, *F*_*Trial1*_(1, 94) = 8.55, *p* = .004, and *F*_*Trial2*_(1, 185.56) = 38.74, *p* < .001, respectively. This two-way interaction in the second trial qualified a significant main effect personality dimension, showing that participants generally made higher judgments for competence (*M* = 4.16, *SE* = .11) than for warmth (*M* = 3.59, *SE* = .11), *F*(1, 185.56) = 7.80, *p* = .006.

We also specified trial-based models with the three fixed factors facial appearance, gender typicality, and personality dimension, all three two-way interactions, and the three-way interaction as well as the above specified random effects in both trials separately. Whereas the three-way interaction did not reach statistical significance in the first trial, *F*(1, 38.99) = 1.33, *p* = .256, it did reach significance in the second, *F*(1, 185.56) = 5.56, *p* = .019.

### Discussion

Replicating the results from Study 1, we found clear and strong evidence for feature-trait associations. Overall, masculine-looking target persons were ascribed more competence and less warmth than feminine-looking persons, independent of gender category information. Again, this was true for both the first and the second trial per person, signaling that this effect does not require comparing persons with each other. As in Study 1, we not only found the hypothesized feature-trait associations in both the typical and the atypical condition, but we even found that effects were stronger in the atypical than in the typical condition. These repeatedly found effects support our assumption that gender category is used as a standard against which atypical members are contrasted. Even though the procedural conditions should have made gender category more salient than in Study 1, gender category was not used to draw stereotypical information from, but as a frame of reference with which the atypical member was compared.

## General discussion

Taken together, results from both studies presented here provide evidence in support of our three hypotheses: Masculine-looking persons are perceived as more competent and less warm than feminine-looking persons (Hypothesis 1, feature-trait associations). This effect of feature-trait associations was not only present for typical and atypical category exemplars (Hypothesis 2, feature-trait associations override category-based stereotyping), it was even more pronounced for atypical than for typical exemplars (Hypothesis 3, contrast effect for atypical exemplars).

Interestingly, the subtle manipulations of facial masculinity/femininity were powerful enough that they were reflected in an absolute (rather than a relative) measure. Moreover, these feature-trait association effects were significant in both studies even if only the first trial per participant were analyzed. These results suggest that no comparison was needed to evaluate a target person differently depending on the degree of masculinity/femininity in his or her face. This impact of facial masculinity/femininity persisted even though much more distinct and likewise visibly perceived information, that is, gender category information was provided. The finding that facial features strongly impacted personality ascriptions fits well into existing literature showing that people base diverse social judgments of others on subtle facial information, for example, about sexual orientation [49], political orientation [50], morality [51], criminality [52], or central personality dimensions [21].

How did gender category information impact how first impressions were formed? Interestingly, the activated gender stereotype did not translate into explicit gender-stereotypical judgments. The lack of category-based gender stereotyping in the two studies presented here and the existence of feature-trait associations at the same time might be explained by differences in the controllability of the two processes [24,25]. Participants might have been aware of their tendency to judge target persons in a gender-stereotypic way and therefore adjusted their judgments for these effects. Feature-trait associations though seem to be a less controlled process, presumably because personality judgments based on faces made automatically [53] and because the influence from facial cues may be monitored less. People may not be aware of this influence or may not consider it as an undue influence but rather as diagnostic individuating information. Literature on social categorization has shown that when individuating information is available, category information is less likely to be used for impression formation [26].

Intriguingly, however, participants did not ignore the gender category. Both studies presented here found evidence that the gender category was used for the judgment but in the opposite way as would be expected from category-based stereotyping. The effect that masculine-looking women were ascribed more competence and less warmth than feminine-looking men (atypical condition) was not solely based on the difference in facial features as this effect tended to be weaker for masculine-looking men compared to feminine-looking women (typical condition). These results suggest that the judgment was shifted away from the respective stereotype relating to the gender category. Such contrast effects to the category suggest that the activated gender stereotype was used as a frame of reference [31]. The masculine-looking woman was excluded from the category of women and therefore contrasted against the group stereotype.

We would like to point out that our method to disentangle extra-facial category-cueing hairstyle information and inner-facial masculinity/femininity and to systematically investigate their impact on impression formation was very subtle. Using a statistical face space approach allowed us to generate a vector that best describes the difference between the average male and female face and to model facial masculinity/femininity in a completely bottom-up data-driven approach. Because the average male and the average female face in our face model differ both regarding featural (e.g., shape of the eyes) as well as configural information (e.g., distance of features from each other), our masculinity/femininity vector contains both featural and configural information. We combined this approach to model facial masculinity/femininity with up-to-date image manipulation techniques, which resulted in facial stimuli that are both highly controlled and natural looking. By manipulating only masculinity/femininity within the same target person without manipulating other facial appearance information (e.g., clothing) we could ensure that any inferences are indeed due to variations in facial masculinity/femininity. Variations in styling, for example, a woman wearing a flowery blouse to create a feminine facial appearance, would have been much more obvious and may have been prone to demand effects.

For two reasons, we are very confident that these effects generalize across faces and participants. Firstly, the vector to model facial masculinity/femininity has been developed based on a set of 200 face scans and has then been applied to a set of totally independent face photographs. So, exactly the same sort and amount of information was manipulated in all faces. This method has been shown to generalize across participants and faces before [16,21,51,52]. Secondly, the statistical method we applied to analyze our data, namely the linear mixed models analyses included random effects for participants and faces. This allowed us to quantify the information explained by the random effects and to show that the effects found generalize across participants and faces.

Extending previous research showing that feminine-looking men and masculine-looking women activate both the male and the female gender stereotype [1,28] and that gender category activates the corresponding gender stereotypes even if category members are atypical [1], we applied this novel approach to manipulate facial stimuli to measure how these two activated gender stereotypes impact impression formation for typical and for atypical category members. Going one step further and assessing how persons are evaluated based on their individual combination of category membership and facial masculinity/femininity seems important because such impressions likely shape behavior in diverse social contexts.

We are only aware of one previous finding showing a contrast effect for category-based stereotyping and an assimilation effect for facial appearance. In a study on prison inmates Blair and colleagues [11] found that although Afrocentric facial features rather than ethnic category membership determined prison sentences, white offenders received more severe sentences than African Americans when controlling for Afrocentric features (and criminal record). The latter reflects a contrast to the stereotype of African Americans being more aggressive than White Americans. The authors explained that, on average, African Americans had more pronounced Afrocentric features than Whites but there was also some overlap. A similar level of Afrocentric features then represented an atypical low level for African-Americans and therefore led to lower sentences, whereas it represented an atypical high level for White Americans and therefore led to higher sentences. In line with these results, our results also suggest that, on the one hand, facial features may directly guide personality inferences, but, on the other hand, that judgments are built by using a standard of comparison from the respective category. Different from this seminal study by Blair and colleagues [25], our studies manipulated facial features orthogonally to category membership. We can therefore rule out any other confounded variables as contributing to the effect.

We acknowledge that the contrast effect due to category membership was rather small in both studies. Maybe more importantly, our results clearly argue against category-based stereotyping *parallel* to feature-trait associations, as this would have implied weaker–not stronger–feature effects when features and category membership have opposite implications.

In line with the inclusion/exclusion framework of Schwarz and Bless [12,31] and with evidence for parallel activation of exclusive gender categories for atypical exemplars from hand movements in the study by Freeman and Ambady [28], we argue that in the case of atypical category exemplars, the activated gender category was used as a standard of reference against which the atypical exemplar was evaluated, leading to a contrast effect. We assume that this mechanism was at work leading to stronger feature effects for atypical than for typical category exemplars. However, one potential alternative explanation would possibly lead to similar findings. One could argue that the same degree of facial masculinity/femininity becomes more salient the more it is perceived to stand in contrast to the respective gender category. The same degree of femininity, thus, would be perceived as more salient in a male face. Due to this enhanced salience, atypical features may more strongly impact personality ascriptions than more typical features. Future studies might focus on whether the contrast between the facial masculinity/femininity and the category information or a contrast from the stereotype drives the typicality by facial masculinity/femininity interaction or whether these two processes go hand in hand.

## Conclusions

The two studies presented here provide evidence that subtle gendered facial appearance information strongly impacts first impressions. In line with the inclusion/exclusion model of stereotyping, these effects are even stronger for atypical than for typical category members. This finding is especially intriguing because facial appearance has been shown to be quite an invalid cue for personality [54,55]. Because facial appearance is the first piece of information available in many situations and because first impressions strongly impact further processing and decision making, these invalid facial appearance cues can have a drastic impact in various applied contexts from personnel selection or political elections to criminal sentencing.

## References

[1] Martin D, Macrae CN. A face with a cue: Exploring the inevitability of person categorization. Eur J Soc Psychol. 2007 5;37(5):806–16.

[2] Willis J, Todorov A. First impressions: Making up your mind after a 100-ms exposure to a face. Psychol Sci. SAGE Publications; 2006 7;17(7):592–8. doi: 10.1111/j.1467-9280.2006.01750.x 16866745 10.1111/j.1467-9280.2006.01750.x

[3] Locke V, Macrae CN, Eaton JL. Is person categorization modulated by exemplar typicality? Soc Cogn. 2005 10;23(5):417–28.

[4] Cloutier J, Macrae N. Who or what are you?: Facial orientation and person construal. Eur J Soc Psychol. 2007 4;37:1298–309.

[5] Armann R, Bülthoff I. Male and female faces are only perceived categorically when linked to familiar identities—And when in doubt, he is a male. Vision Res. Elsevier Ltd; 2012 5;63:69–80. doi: 10.1016/j.visres.2012.05.005 22595743 10.1016/j.visres.2012.05.005

[6] Hoss R, Ramsey JL, Griffin AM, Langlois JH. The role of facial attractiveness and facial masculinity/femininity in sex classification of faces. Perception. 2005 12;34(12):1459–74. doi: 10.1068/p5154 16457167 10.1068/p5154 PMC1368665

[7] O’Toole AJ, Deffenbacher K, Valentin D, McKee K, Huff D, Abdi H. The perception of face gender: The role of stimulus structure in recognition and classification. Mem Cognit. 1998 1;26(1):146–60. 9519705 10.3758/bf03211378

[8] Oosterhof NN, Todorov A. The functional basis of face evaluation. Proc Natl Acad Sci. 2008 8;105(32):11087–92. doi: 10.1073/pnas.0805664105 18685089 10.1073/pnas.0805664105 PMC2516255

[9] Deaux KLF, Lafrance M. Gender In: Gilbert D, Fiske ST, Lindzey G, editors. The Handbook of Social Psychology. 4th ed. New York: Random House; 1998 p. 788–827.

[10] Zebrowitz L. Ecological and social approaches to face perception In: Calder AJ, Rhodes G, Johnson MH, Haxby JV, editors. The Oxford Handbook of Face Perception. Oxford: Oxford University Press; 2011 p. 31–50.

[11] Blair IV, Judd CM, Chapleau KM. The influence of afrocentric facial features in criminal sentencing. Psychol Sci. 2004 10;15(10):674–9. doi: 10.1111/j.0956-7976.2004.00739.x 15447638 10.1111/j.0956-7976.2004.00739.x

[12] Schwarz N, Bless H. Mental construal processes: The inclusion/exclusion model In: Stapel D, Suls J, editors. Assimilation and Contrast in Social Psychology. Psychology Press; 2007 p. 1–32.

[13] Brebner JL, Martin D, Macrae CN. Dude looks like a lady: Exploring the malleability of person categorization. Eur J Soc Psychol. 2009 2;39(6):1109–19.

[14] Cellerino A, Borghetti D, Sartucci F. Sex differences in face gender recognition in humans. Brain Res Bull. 2004 7;63(6):443–9. doi: 10.1016/j.brainresbull.2004.03.010 15249109 10.1016/j.brainresbull.2004.03.010

[15] Cheng YD, O’Toole AJ, Abdi H. Classifying adults’ and children’s faces by sex: Computational investigations of subcategorical feature encoding. Cogn Sci. 2001 10;25(5):819–38.

[16] Walker M, Vetter T. Portraits made to measure: Manipulating social judgments about individuals with a statistical face model. J Vis. 2009 10;9(11):1–13. 10.1167/9.11.12 20053075

[17] Blanz V, Vetter T. A morphable model for the synthesis of 3D faces. Proc 26th Annu Conf Comput Graph Interact Tech. 1999 7;19(7):187–94.

[18] Little AC, Jones BC, DeBruine LM, Dunbar RIM. Accuracy in discrimination of self-reported cooperators using static facial information. Pers Individ Dif. 2013 3;54(4):207–512.

[19] Paysan P, Knothe R, Amberg B, Romdhani S, Vetter T. A 3D face model for pose and illumination invariant face recognition In: Advanced Video and Signal Based Surveillance. IEEE; 2009 9 p. 296–301.

[20] Vetter T, Walker M. Computer-generated images in face perception In: Calder AJ, Rhodes G, Johnson MH, Haxby JV, editors. The Oxford Handbook of Face Perception. Oxford: Oxford University Press; 2011 p. 387–400.

[21] Walker M, Vetter T. Changing the personality of a face: Perceived Big Two and Big Five personality factors modeled in real photographs. J Pers Soc Psychol. 2016 4;110(4):609–24. doi: 10.1037/pspp0000064 26348599 10.1037/pspp0000064

[22] Fiske ST, Neuberg SL. A continuum of impression formation, from category-based to individuating processes: Influences of information and motivation on attention and interpretation In: Zanna MP, editor. Advances in Experimental Social Psychology. New York: Academic Press; 1990 p. 1–74.

[23] Fiske ST, Cuddy AJC, Glick P, Xu J. A model of (often mixed) stereotype content: Competence and warmth respectively follow from perceived status and competition. J Pers Soc Psychol. 2002 6;82(6):878–902. 12051578

[24] Blair IV, Judd CM, Sadler MS, Jenkins C. The role of afrocentric features in person perception: Judging by features and categories. J Pers Soc Psychol. 2002 7;83(1):5–25. 12088132

[25] Blair IV, Judd CM, Fallman JL. The automaticity of race and afrocentric facial features in social judgments. J Pers Soc Psychol. 2004 12;87(6):763–78. doi: 10.1037/0022-3514.87.6.763 15598105 10.1037/0022-3514.87.6.763

[26] Kunda Z, Thagard P. Forming impressions from stereotypes, traits, and behaviors: A parallel-constraint-satisfaction theory. Psychol Rev. 1996 4;103(2):284–308.

[27] Chen M, Bargh JA. Nonconscious behavioral confirmation processes: The self-fulfilling consequences of automatic stereotype activation. J Exp Soc Psychol. 1997 9;33(5):541–560.

[28] Freeman JB, Ambady N. Motions of the hand expose the partial and parallel activation of stereotypes. Psychol Sci. 2009 10;20(10):1183–8. doi: 10.1111/j.1467-9280.2009.02422.x 19686295 10.1111/j.1467-9280.2009.02422.x

[29] Bless H, Schwarz N, Bodenhausen GV, Thiel L. Personalized versus generalized benefits of stereotype disconfirmation: Trade-offs in the evaluation of atypical exemplars and their social groups. J Exp Soc Psychol. 2001 9;37(5):386–97.

[30] Schwarz N, Bless H. Constructing reality and its alternatives: Assimilation and contrast In: Martin LL, Tesser A, editors. The Construction of Social Judgments. Hillsdale, NJ: Lawrence Erlbaum Associates, Inc.; 1992 p. 217–45.

[31] Bless H, Schwarz N. Mental construal and the emergence of assimilation and contrast effects: The inclusion/exclusion model In: Zanna MP, editor. Advances in Experimental Social Psychology, Vol 42 San Diego, CA: Elsevier; 2010 p. 319–73.

[32] Wänke M, Bless H, Wortberg S. Der Einfluss von „Karrierefrauen”auf das Frauenstereotyp: Moderatoren von Stereotypenänderung und Subtyping. (The impact of career women on gender stereotypes: moderators and subtyping). Zeitschrift für Sozialpsychologie. 2003; 34:187–96.

[33] Biernat M. Toward a broader view of social stereotyping. Am Psychol. 2003 12;58(12): 1019–27. doi: 10.1037/0003-066X.58.12.1019 14664690 10.1037/0003-066X.58.12.1019

[34] Little AC, DeBruine LM, Feinberg DR, Perrett DI. Men’s strategic preferences for femininity in female faces. Br J Psychol. 2014 8;105(3):364–81. doi: 10.1111/bjop.12043 25040006 10.1111/bjop.12043

[35] Questback GmbH. EFS Survey, Version 10.5. Köln: Questback GmbH; 2015 Available from: www.unipark.ch

[36] Westfall J. PANGEA: Power ANalysis for GEneral Anova designs. 2015. Available from: http://jakewestfall.org/publications/pangea.pdf

[37] Phillips PJ, Wechsler H, Huang J, Rauss PJ. The FERET database and evaluation procedure for face-recognition algorithms. Image Vis Comput. 1998 4;16(5):295–306.

[38] Walker M, Jiang F, Vetter T, Sczesny S. Universals and cultural differences in forming personality trait judgments from faces. Soc Psychol Personal Sci. 2011 5;2(6):609–17.

[39] Schneider-Düker M, Kohler A. Die Erfassung von Geschlechtsrollen: Ergebnisse zur deutschen Neukonstruktion des Bem Sex-Role Inventory. Diagnostica. 1988;34(3):256–70.

[40] Bem S. The measurement of psychological androgyny. J Consult Clin Psychol. 1974 4;42(2):155–62. 4823550

[41] Spence JT, Buckner CE. Instrumental and expressive traits, trait stereotypes, and sexist attitudes: What do they signify? Psychol Women Q. 2000 3;24(1):44–53.

[42] Martin CL. A ratio measure of sex stereotyping. Pers Soc Psychol. 1987 3;52(3):489–99.

[43] Judd CM, Westfall J, Kenny DA. Treating stimuli as a random factor in social psychology: A new and comprehensive solution to a pervasive but largely ignored problem. J Pers Soc Psychol. 2012 7;103(1):54–69. doi: 10.1037/a0028347 22612667 10.1037/a0028347

[44] Bates D, Maechler M, Bolker B, Walker S. lme4: Linear mixed-effects models using Eigen and S4. R package version 1.0–5. 2014.

[45] R Core Team. R: A Language and Environment for Statistical Computing. R Foundation for Statistical Computing, Vienna, Austria; 2014.

[46] Barr DJ, Levy R, Scheepers C, Tily HJ. Random effects structure for confirmatory hypothesis testing: Keep it maximal. J Mem Lang.2013 4;68(3):255–78. 10.1016/j.jml.2012.11.001 PMC3881361 24403724

[47] Kuznetsova A, Brockhoff PB, Christensen RHB. lmerTest: Tests in Linear Mixed Effects Models. R package version 2.0–20. 2015.

[48] Gmuer M. KMGI–Konstanzer Managergeschlechtsrolleninventar: Ein Instrument zur Ermittlung der Wirksamkeit von Geschlechtsrollenstereotypen in der Personalauswahl. Forschungsbericht Nr. 1 des Lehrstuhls für Management der Universität Konstanz. 1991.

[49] Rule NO, Ambady N. Brief exposures: Male sexual orientation is accurately perceived at 50ms. J Exp Soc Psychol. 2008 7;44(4):1100–5.

[50] Samochowiec J, Wänke M, Fiedler K. Political ideology at face value. Soc Psychol Personal Sci. 2010 7;1(3):206–13.

[51] Rudert SC, Reutner L, Greifeneder R, Walker M. Faced with exclusion: Perceived facial warmth and competence influence moral judgments of social exclusion. J Exp Soc Psychol. 2017 1;68:101–12.

[52] Funk F, Walker M, Todorov A. Modelling perceptions of criminality and remorse from faces using a data-driven computational approach. Cogn Emot. 2016 9:1–13. 10.1080/02699931.2016.1227305 27603691

[53] Ballew CC, Todorov A. Predicting political elections from rapid and unreflective face judgments. Proc Natl Acad Sci. 2007 11;104(46):17948–53. doi: 10.1073/pnas.0705435104 17959769 10.1073/pnas.0705435104 PMC2084277

[54] Pound N, Penton-Voak IS, Brown WM. Facial symmetry is positively associated with self-reported extraversion. Pers Individ Dif. 2007 10;43(6):1572–82.

[55] Shevlin M, Walker S, Davies MNO, Banyard P, Lewis CA. Can you judge a book by its cover? Evidence of self–stranger agreement on personality at zero acquaintance. Pers Individ Dif. 2003 10;35(6):1373–83.

